# State of the Globe: Rabies is Still Rampant and Needs Action

**DOI:** 10.4103/0974-777X.68523

**Published:** 2010

**Authors:** Nicholas Johnson

**Affiliations:** *Rabies and Wildlife Zoonoses Group, Veterinary Laboratories Agency – Weybridge, Woodham Lane, Addlestone, Surrey, KT15 3NB*

Rabies is endemic within the dog population of both Africa and Asia, and there is little prospect that this will change in the near future. The estimated number of human deaths each year due to rabies infection is regularly quoted around the 50,000 mark. It could be more or it could be less, although the informed consensus is that it is much more. This uncertainty is due to the absence of surveillance or reporting in many countries of the world. In the absence of reliable information, how can countries assess the extent of the problem and implement a solution? Beyond the headline figures are the consequences of rabies, the many deaths, often of young children, and the fear of disease ensuing from a dog bite. By contrast, there also prevails a lot of ignorance on the best course of action to take following biting events. For those living in rabies-endemic regions or visitors to such areas, the disease is a major public health problem and one that requires more information before effective action can be taken.

Epidemiological information can be generated by assessing the numbers of people seeking treatment following an animal bite.[1] The article by Humphrey Mazigo and co-workers[2] in this issue illustrates this. Firstly, what is the extent of the problem? The authors have reported the number of people receiving post-exposure prophylaxis (PEP) in response to biting incidents in a single region of Tanzania over a five-year period. Their report suggests a mean annual incidence of 58 cases per 100,000 of population, with the majority being children (55%). Secondly, which animals are causing biting incidents? In the study, over 95% of incidents occurred due to dog bites. Other species included the domestic cat, spotted hyena and black-backed jackal. Whilst wildlife can act as a reservoir for rabies and on occasion transmit disease to humans following a bite, dogs are the main problem; and resources need to be focused on solving the problem of rabies in dogs. Finally, what is the compliance rate for a full course of vaccination injections within this population? The authors assessed the compliance of those receiving the full course of three inoculations. Sadly, of the 767 individuals monitored, over 550 did not return to receive a second or third inoculation. This level of noncompliance reflects either a unwillingness to pay for a full course of treatment or a lack of understanding of the potential outcome of not completing the course. Both reasons undermine the effectiveness of providing therapy in resource-poor countries.

What action is currently being taken? A number of charities under the umbrella organization Alliance for Rabies Control (http://www.rabiescontrol.net/) are raising awareness about rabies, particularly through publication of useful information and promotion of World Rabies Day (September 10). Furthermore, a number of charities and experts are attempting to capture those actions that are required at all levels of government in the form of a blueprint that could form an effective rabies control and elimination plan. Details will be available shortly (http://www.rabiesblueprint.com/). Coordinated public health campaigns can be effective at controlling dog rabies as evidenced by the example of Latin America, where strategic decisions were made jointly by countries to address rabies control.[3] These clearly need to include education about rabies and the appropriate response to a bite from a potentially rabid animal. This can include simple, inexpensive actions such as thorough wound washing to obtaining appropriate post-exposure vaccination. The World Health Organization recommends the use of cell-culture–produced vaccines rather than nerve tissue–derived vaccine, although problems including lack of availability, cost and compliance continue to hinder its effective application in many regions of the world. Yet it is the control of rabies in dogs that has proven the most effective way of preventing human infection. The challenges faced in controlling rabies in many countries are extensive, and there are as many reasons for inaction, although none of these are insurmountable.[4] Ultimately it will require action from countries themselves and those that govern them. Studies such as that described in this issue will enable the governments of rabies-endemic countries to recognize that there is a problem. Such studies also highlight the burden both financial and to health and development that rabies causes. Finally they quantify the potential benefits of controlling rabies through action.
